# A Static-to-Temporal Framework for Interpretable Camera Lens Soiling Severity Estimation in Autonomous Driving

**DOI:** 10.3390/s26113533

**Published:** 2026-06-03

**Authors:** Fan Yang, Xingyu Duan, Fan Li, Luolin Zhang

**Affiliations:** 1 State Key Laboratory of Advanced Design and Manufacturing Technology for Vehicle, Hunan University, Changsha 410082, China; ywyf@hnu.edu.cn (F.Y.); brycewg@hnu.edu.cn (L.Z.); 2 College of Intelligence Science and Technology, National University of Defense Technology, Changsha 410073, China; duanxingyu19@nudt.edu.cn

**Keywords:** camera lens soiling, severity estimation, dual-head learning, stable diffusion, temporal stabilization, autonomous driving

## Abstract

Camera lens soiling can severely degrade visual perception in autonomous driving, making reliable soiling severity estimation essential for camera-health monitoring and downstream perception safety. However, existing methods mainly rely on area-based indicators or frame-wise predictions, which insufficiently account for opacity differences, spatial importance, and temporal stability in continuous video streams. To address these limitations, this paper proposes a static-to-temporal soiling framework for camera-soiling severity estimation. First, we propose a Structured Dual-Head Static Model (SDSM) that jointly predicts tile-level four-class soiling distributions and an image-level severity score. The model is coupled with an explicit Structured Severity Score that aggregates local predictions through opacity-aware, spatial, and dominance-related components. Second, to alleviate the scarcity of real temporal soiling data, we construct a Two-Stage Stable Diffusion (TS-SD) pipeline and use the resulting SD-Seq data as mechanism supervision for temporal learning rather than direct single-frame strong supervision. Finally, we introduce a structure-constrained adaptive EMA Module to improve temporal stability while preserving the original single-frame severity scale. Experiments on WoodScape, External Test, and OccNuScenes-Dirt show strong cross-domain severity estimation performance, including a cluster-level Spearman correlation of 0.7876 on External Test. The temporal module further reduces Jitter (MAD) by 51.5%. These results demonstrate an interpretable, cross-domain, and deployment-friendly solution for camera-soiling assessment.

## 1. Introduction

Autonomous driving and active safety systems rely heavily on robust environmental perception, and surround-view cameras play a crucial role in providing visual information [1]. For mass-produced surround-view systems, these cameras continuously supply visual input of road scenes [2], and their imaging quality directly affects the effectiveness of downstream perception tasks such as lane line detection and object recognition [3,4]. However, because most cameras are exposed to various soiling such as dust, mud, water stains, and oil [5], captured images may suffer from blur, contrast reduction, and partial occlusion, which in turn degrade the reliability of downstream perception tasks. A key engineering challenge is therefore to estimate lens-soiling severity in a reliable and real-time manner so that the resulting severity signal can support system alarms, camera availability assessment, and downstream functional adaptation [6] under practical in-vehicle deployment constraints.

Existing methods such as SoilingNet [7] and subsequent grid-based soiling modeling [8] approaches show that local soiling coverage offers a practical compromise between performance and deployability. However, these methods mainly describe coverage extent rather than structured visual degradation. On the temporal side, temporal consistency has been recognized as important, yet many existing solutions remain frame-based or depend on heavy video models that are difficult to deploy on edge platforms.

In addition to these modeling limitations, in-vehicle lens-soiling assessment is also constrained by data availability. Currently, real-world temporal soiling data with high-quality annotations are scarce. Publicly available datasets mainly support static supervision. WoodScape [9] illustrates this limitation well. It provides high-quality pixel-level annotations for static severity modeling but offers too few consecutive frames under the same soiling state for direct temporal supervision. Collecting large-scale real-world temporal soiling data with fine-grained annotations is costly and still unlikely to provide sufficient long-tail coverage. Recent work has started to explore generative augmentation for lens-soiling samples. For instance, methods such as Let’s Get Dirty [10] have demonstrated the potential of synthesizing diverse soiling patterns for data augmentation.

Taken together, the above limitations reveal that camera-soiling severity estimation is not only a problem of detecting soiling regions but also a problem of defining a reliable severity scale for deployment-oriented monitoring. A simple area-based score may be sufficient to describe how much of the image is covered, but it cannot distinguish whether the covered regions are transparent stains or opaque mud, whether they appear in task-critical regions, or whether a small but locally dominant occlusion causes substantial perceptual degradation. Meanwhile, video-stream deployment introduces a different requirement: the estimated severity should remain temporally stable when the lens state is physically unchanged, even though the road background, illumination, and moving objects vary continuously. These requirements are difficult to satisfy with either purely frame-based models or heavy video architectures, especially because real-world temporal soiling data with fine-grained annotations remain scarce.

Motivated by this gap, this paper aims to establish a unified static-to-temporal soiling framework with three design objectives. First, the static severity representation should be interpretable and should explicitly encode opacity-dependent degradation, spatial importance, and locally dominant soiling patterns rather than relying only on contaminated area. Second, the learned severity scale should be transferable across domains and should remain consistent when moving from strongly annotated static data to weakly supervised external video data. Third, temporal stabilization should suppress non-physical short-term jitter while maintaining the static severity anchor, rather than replacing it with a separate video model. These objectives lead to a framework in which an interpretable static severity ruler is first constructed, synthetic data are used only for temporal mechanism supervision, and a lightweight adaptive filtering module is trained on top of the frozen static anchor.

**Figure 1:**
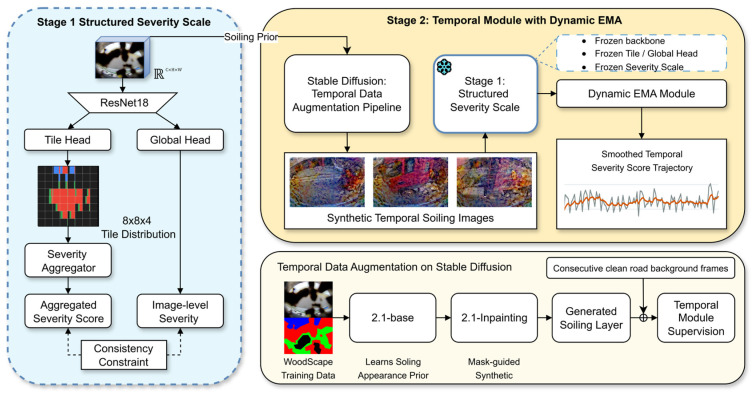
Overall framework of the proposed static-to-temporal soiling framework.

The main contributions of this work are as follows and illustrated in Figure 1. First, we propose an interpretable Structured Dual-Head Static Model (SDSM) for camera lens soiling severity estimation. Instead of treating severity as an area-only statistic, the proposed Structured Severity Score decomposes visual degradation into opacity-aware coverage, spatial importance, and local dominance. This score is calculated by the Severity Aggregator embedded in the SDSM, where tile-level distributions and image-level severity prediction are jointly learned under a consistency constraint. Second, we analyze the limitations of using Stable Diffusion for direct soiling augmentation and utilize it as a source of temporal mechanism supervision, thereby establishing a synthetic soiling sequence paradigm suitable for temporal learning. Third, we introduce a recursively constrained temporal module that reduces output jitter while retaining the established severity ordering. Finally, we provide a multi-protocol evaluation of the proposed framework. Through cluster-level evaluation and bootstrap analysis, we further demonstrate that the resulting stabilization gains are broadly consistent across clusters and statistically reliable.

The pipeline has three stages: (1) the SDSM establishes an interpretable severity scale from tile-level soiling distributions and image-level prediction; (2) TS-SD constructs SD-Seq for temporal learning; (3) the adaptive EMA Module is trained on the frozen static anchor to improve temporal stability while preserving the original severity scale.

## 2. Related Work

Recent research on in-vehicle camera lens soiling and severity modeling can be broadly grouped into three directions: soiling detection and quantification, synthetic data augmentation, and temporal modeling and stabilization. Existing work has laid important groundwork in local coverage modeling, soiling sample expansion, and output smoothing. However, few studies address structured severity representation, controllable synthetic supervision, and lightweight temporal stabilization within a unified framework.

### 2.1. Soiling Detection and Quantification

In camera soiling detection, existing studies move from image-level recognition toward fine-grained local modeling. A representative line of work is the SoilingNet and TiledSoilingNet series developed by Valeo, which advances surround-view soiling detection toward tile-level coverage modeling [7,8,11]. TiledSoilingNet directly regresses the coverage of each soiling type within a tile rather than predicting only the dominant class, making it better suited to mixed-soiling cases within the same tile. In parallel, datasets such as WoodScape incorporate soiling into the multi-task perception setting of autonomous driving, extending evaluation from standard forward-view images to fisheye surround-view scenarios that are more relevant to production systems [12,13]. Building on these efforts, later studies further explore region-level representations such as tile-based class prediction, coverage estimation, local region localization, and segmentation of localized transparent stains and non-uniform soiling patterns.

However, these representations remain closer to local coverage statistics and category distributions than to a structured description of visual degradation [14]. From a broader reliability perspective, camera soiling can also be regarded as a camera image degradation or sensor-failure-related condition in autonomous driving. Recent surveys on sensor failures emphasize that camera image failures and environmental disturbances can affect perception reliability and require system-level mitigation strategies such as redundancy, calibration, and sensor fusion [15]. Therefore, for lens-soiling assessment, the remaining challenge is not only to describe local soiling coverage but also to map tile-level soiling statistics to a global severity representation that reflects transparency, occlusion intensity, spatial importance, and locally dominant soiling while preserving local interpretability.

### 2.2. Synthetic Soiling Generation and Data Augmentation

Because real-world soiling data are costly to collect and annotate, and long-tail coverage is difficult to achieve, synthetic data augmentation has emerged as an important direction in this field. Early work mainly relies on GAN-based domain transfer and mask-guided generation, where soiled images and corresponding soiling masks are synthesized jointly to reduce manual annotation effort. Let’s Get Dirty is a representative work along this line. It uses a GAN-based synthesis to generate previously unseen soiling patterns for augmentation. More importantly, it explicitly highlights that lens soiling differs from generic adverse weather because it often remains static or evolves only slowly over time. This observation underscores the task-specific importance of temporal consistency.

Another representative line is programmatic modeling and physics-inspired soiling synthesis [16]. These methods are attractive because the generation process is interpretable, the placement of soiling can be controlled, and annotations can be obtained simultaneously. Their limitations, however, lie in strong modeling assumptions, reduced realism for complex appearances, and limited scalability in generating diversity samples at large scale, often with higher manual design cost. More broadly, synthetic data in autonomous driving are known to suffer from a synthetic-to-real domain gap, despite their value for controllability, scalability, and annotation efficiency [17]. Recent controlled degradation datasets, such as Occluded nuScenes, further provide parameterized and reproducible sensor occlusions across camera, radar, and LiDAR modalities for robustness evaluation under partial sensor failures and environmental interference [18]. This synthetic-to-real domain gap becomes more challenging for temporal lens-soiling modeling, because real contamination may evolve through accumulation, motion, adhesion, partial removal, or cleaning-related changes. Existing physics-based or controlled degradation datasets mainly support controllable degradation generation and reproducible robustness evaluation, rather than fully modeling the temporal evolution of lens contamination. Therefore, in this work, synthetic soiling sequences are used cautiously as mechanism-oriented temporal supervision rather than as a substitute for real temporal soiling videos.

More recently, diffusion models have been adopted for long-tail scenario synthesis in autonomous driving [19]. Existing work based on Stable Diffusion or ControlNet [20] mainly focuses on the driving scene generation, adverse-weather editing, and other forms of structurally controlled scene variations. These methods show strong potential for producing natural visual degradation effects—such as rain, snow, and fog, while preserving road structure, semantic layout, and depth relations [21,22]. However, visually plausible degradation does not necessarily imply reliable soiling severity semantics or realistic temporal dynamics. This limitation is consistent with the broader synthetic-to-real gap in autonomous-driving synthetic data. For the present task, this limitation motivates our repositioning of synthesis from direct frame-level supervision to temporal mechanism supervision [23].

### 2.3. Temporal Smoothing and Modeling

Lens-soiling assessment is typically performed on continuous video streams, yet many existing methods still operate frame by frame. As a result, predictions become sensitive to background texture, illumination changes, moving objects, and other scene variations, leading to unstable soiling scores or frequent state switching. Unlike external weather conditions such as rain, snow, and fog, lens soiling adheres to the lens surface and usually exhibits distinct temporal consistency over short time windows. This task-specific temporal consistency is also emphasized in Let’s Get Dirty. Accordingly, existing methods can be categorized into two types: traditional smoothing and deep temporal modeling.

Traditional methods include low-pass filtering, probabilistic filtering, and exponential moving averages (EMAs). These methods are computationally efficient and well suited to real-time, on-board deployment. EMA is a representative example: it stabilizes predictions by assigning exponentially decaying weights to historical observations. Its limitation, however, is that a fixed α must trade off steady-state smoothing against rapid response to genuine changes, which often leads to lag.

A second line of work uses learnable temporal models developed for efficient video understanding. Recent video models such as MoViNets [24], MViTv2 [25], and Video Swin Transformer [26] demonstrate strong capability in spatio-temporal feature extraction through temporal convolution, multi-scale attention, or hierarchical windowing. These advances provide useful methodological references for video-based soiling assessment. However, such deep temporal models typically require more real-world temporal data and incur higher computational costs.

A more relevant direction for the present task comes from differentiable filtering and neural state estimation. Representative studies such as KalmanNet [27] and Differentiable Particle Filters [28] show that filtering can be combined with learning to improve robustness to noisy observation and non-linear dynamics while preserving structural priors in state updates. This line of work suggests that temporal stability does not necessarily require heavy temporal networks. This idea directly motivates our later use of a lightweight, structure-constrained temporal stabilization module.

## 3. Materials and Methods

This section describes the datasets, model design, temporal supervision strategy, and evaluation protocols used in the proposed soiling framework. We first establish the static foundation through SDSM on the strongly annotated WoodScape dataset then construct SD-Seq from the TS-SD pipeline for temporal supervision and finally introduce a structure-constrained adaptive EMA Module on top of the frozen static ruler to improve output stability while preserving the severity scale. Evaluation is performed using dataset-specific metrics and statistical validation schemes that account for differences in annotation format and temporal correlation.

### 3.1. Data Resources Used in the Framework

#### 3.1.1. WoodScape: Primary Strongly Annotated Dataset

**Figure 2:**
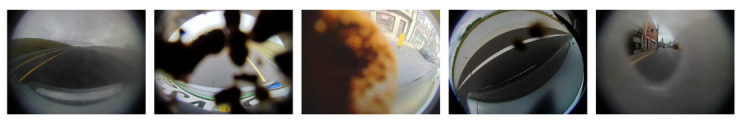
Representative training samples from the WoodScape dataset.

The static model in this work is trained entirely on the WoodScape surround-view soiling dataset. As shown in Figure 2, WoodScape contains diverse soiling patterns, ranging from mild transparent stains to large-area opaque occlusions, which makes it suitable for static severity modeling. Each image is provided with high-quality pixel-level semantic annotations. In particular, the four-class label scheme—clean, transparent, semi-transparent, and opaque—captures not only soiling presence but also opacity-dependent differences in occlusion, which are essential for severity estimation. We use 3200, 800, and 1000 images for training, validation, and testing, respectively, for a total of 5000 static samples.

#### 3.1.2. External Test and OccNuScenes-Dirt: Independent External Evaluation Sets

In addition to WoodScape, this paper uses two independent external evaluation sets that are not involved in model training. Although they differ in severity definition and evaluation role, together they form the complementary external evidence for generalization.

External Test serves as the primary real-world benchmark and is used to evaluate cross-domain soiling severity discrimination and ranking. It was obtained through an approved collaboration with a commercial-vehicle autonomous-driving company and consists of real in-vehicle video frames collected during normal operation of hundreds of commercial trucks and freight vehicles across multiple regions in several provinces. The dataset covers diverse road-use conditions, including public roads, construction-site roads, and other daily operating scenarios.

**Figure 3:**
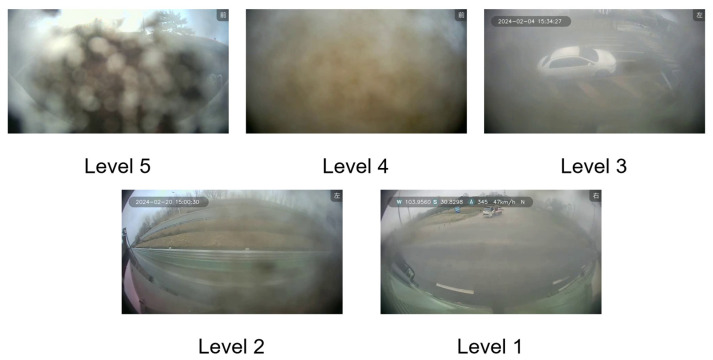
Representative examples from the External Test across severity levels 1–5.

Unlike WoodScape, External Test does not provide pixel-level or tile-level strong annotations. Instead, it is provided with five-level weak visual severity labels, where Level 1 denotes the lightest overall lens soiling and Level 5 denotes the most severe degradation. Representative samples of External Test are shown in Figure 3, where the Chinese labels in the upper-right corner of each image indicate the corresponding mounting position of the in-vehicle camera. The dataset contains 522 temporal clusters formed by consecutive frames, with 50,458 images in total and about 97 frames per cluster on average. From Level 1 to 5, the numbers of frames are 11,384, 13,665, 8596, 13,752, and 3061, respectively, and the corresponding numbers of clusters are 148, 152, 85, 120, and 17. Because adjacent frames are strongly temporally correlated, a cluster-level evaluation protocol is adopted to mitigate the bias that would arise from treating adjacent frames as independent observations.

**Figure 4:**
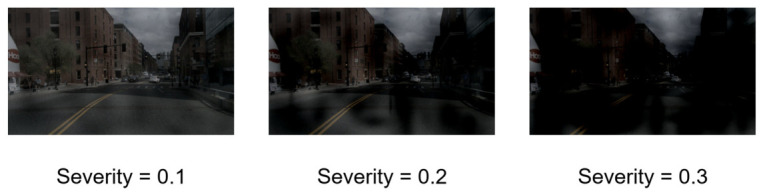
Representative OccNuScenes-Dirt examples with severity values of 0.1, 0.2, and 0.3.

OccNuScenes-Dirt serves as a supplementary controlled benchmark. As shown in Figure 4, its severity labels are defined by generation parameters at three progressive levels, 0.1, 0.2, and 0.3, and the dataset provides same-background triplets. This setting enables us to test whether the model responds monotonically to controlled severity increments.

### 3.2. Static Foundation: Structured Dual-Head Model

The SDSM forms the static foundation of the proposed framework. It jointly learns tile-level soiling distributions and image-level severity prediction and the Structured Severity Score defines the explicit aggregation rule from local coverage statistics to global severity. Within this framework, the score s serves as the area-only reference, whereas $S_{full\text{-}wgap\text{-}alpha50}$ denotes the main static model adopted in the subsequent experiments and temporal extension.

#### 3.2.1. Architecture of the SDSM

SDSM is built on a ResNet18 [29] backbone. The tile head predicts tile-level four-class soiling coverage and produces the aggregated severity score $S_{agg}$ through the Severity Aggregator, whereas the global head directly regresses the image-level severity prediction $\hat{S}$. This dual-head design combines local spatial interpretability with image-level severity modeling. The two heads are further coupled by a consistency loss $L_{\mathrm{cons}}$, which constrains $\hat{S}$ and $S_{agg}$ to remain in the same severity space. Importantly, $S_{agg}$ is not an arbitrarily learned latent variable; it is explicitly obtained by aggregating tile-level distributions according to the predefined score formulation. Under the consistency constraint, this anchors the global prediction to local structural information and provides a structured explanation for global soiling severity prediction.

Given the real-time and deployment constraints of in-vehicle edge platforms, ResNet18 is selected as the backbone. It uses a simple and mature operator set and does not depend on components such as Swish, GELU, or Attention-based modules, which are often less favorable on early-generation NPUs or DSPs. ResNet18 is also compatible with common automotive accelerators and standard quantization workflows, making it suitable for standalone deployment in non-core perception sub-tasks.

#### 3.2.2. Structured Severity Score Formulation

The WoodScape samples used by SDSM consist of RGB images, tile-level four-class coverage labels, and global severity labels derived from the same severity score definitions. All fisheye inputs are resized to a resolution of 640 × 480 without undistortion. Each image is partitioned into an 8 × 8 grid, and the semantic segmentation masks are used to compute the pixel ratios of the four classes—clean, transparent, semi-transparent, and opaque—within each tile. Let $p_{ij}^{c}$ denote the pixel ratio of class *c* in tile (i, j), where *c* ∈ {0, 1, 2, 3} and $\sum_{c}p_{ij}^{c}=1$. The local severity representation $s_{ij}$ is then obtained by mapping the tile-level class distribution through the class weight $w_{c}=[0.0,\;0.33,\;0.66,\;1.0]$. The global severity label is finally aggregated from these local representations according to the Structured Severity Score defined below.

(1) $$p_{ij}=[p_{ij}^{\left(0\right)},\;p_{ij}^{\left(1\right)},\;p_{ij}^{\left(2\right)},\;p_{ij}^{\left(3\right)}]$$

(2) $$p_{ij}^{c}\in \left[0,\;1\right],\;\;\;\;c\in \{0,\;1,\;2,\;3\}$$

(3) $$s_{ij}=\sum_{c=0}^{3}w_{c}p_{ij}^{c}$$

The global soiling severity $S_{agg}$ is defined as the Structured Severity Score aggregated from local coverage statistics. Because different soiling categories contribute differently to visual degradation, $S_{agg}$ is decomposed into three complementary components based on the local severity field $s_{ij}$. Their aggregation weights are set to $\beta =\left[\beta_{op},\;\beta_{sp},\;\beta_{dom}\right]$, with default values of [0.6,0.3,0.1].

(4) $$S_{agg}=\beta_{op}S_{op}+\beta_{sp}S_{sp}+\beta_{dom}S_{dom}$$

$S_{op}$ denotes opacity-aware coverage, i.e., the average occlusion strength after accounting for category-dependent transparency. The class weight $w_{c}$ distinguishes the visual degradation caused by transparent, semi-transparent, and opaque soiling under the same coverage extent. This term therefore captures the fact that equal area does not imply equal severity.

(5) $$S_{op}=\frac{1}{HW}\sum_{i=1}^{H}\sum_{j=1}^{W}s_{ij}$$

$S_{sp}$ represents spatial importance. In this work, $W_{\mathrm{ij}}$ is implemented as a Gaussian spatial map, assigning larger weights to the central image region and smaller weights to peripheral regions. This choice is used as a general spatial prior for road-scene fisheye images, where peripheral regions are often more distorted and may contribute less to major downstream tasks. The framework, however, does not require the spatial map to be fixed. For task-specific deployment, $W_{\mathrm{ij}}$ can be reshaped according to the perception target and camera layout, such as emphasizing lower image regions for pedestrian-oriented tasks or other task-critical regions in different vehicle scenarios.

(6) $$S_{sp}=\sum_{i=1}^{H}\sum_{j=1}^{W}W_{ij}s_{ij}$$

(7) $$\sum_{i=1}^{H}\sum_{j=1}^{W}W_{ij}=1$$

$S_{dom}$ models local dominance and captures the disproportionate influence of local severe soiling on global visual degradation. To emphasize dominant tiles, we apply softmax pooling to local severity field $s_{ij}$, with softmax temperature T = 0.25, yielding the dominance weights $a_{ij}$. The resulting raw dominance score before transparent discount is denoted by $S_{dom}^{raw}$.

(8) $$a_{ij}=\frac{exp(\frac{s_{ij}}{T})}{\sum_{m=1}^{H}\sum_{n=1}^{W}\exp\left(\frac{s_{mn}}{T}\right)}$$

(9) $$S_{dom}^{raw}=\sum_{i=1}^{H}\sum_{j=1}^{W}a_{ij}s_{ij}$$

Because transparent soiling typically causes weaker visual degradation than semi-transparent or opaque soiling under equivalent coverage conditions, a transparent discount is introduced into the dominance term. Let $r_{ij}^{tr}$ denote the proportion of transparent soiling within all soiling pixels in tile (i, j) and let $r^{tr}$ denote its dominance-weighted global counterpart. The complete dominance term is then written as:

(10) $$r^{tr}=\sum_{i=1}^{H}\sum_{j=1}^{W}a_{ij}r_{ij}^{tr}$$

(11) $$r_{ij}^{tr}=\frac{p_{ij}^{\left(1\right)}}{p_{ij}^{\left(1\right)}+p_{ij}^{\left(2\right)}+p_{ij}^{\left(3\right)}+\varepsilon }$$

(12) $$S_{dom}=\left(1-\eta r^{tr}\right)S_{dom}^{raw}$$

where ε is a small constant for numerical stability and is set to 1 × 10^−6^, and η controls the strength of the transparent discount and is set to 0.9. Therefore, η reduces the dominance contribution when the locally dominant soiling is mainly transparent.

The Structured Severity Score is fully specified by the class-weight vector $w_{c}$ and the aggregation-weight vector β. In the main static model $S_{full\text{-}wgap\text{-}alpha50}$, the overall score is kept unchanged, while the two weight sets are rebalanced to ***w****_c_* = [0, 0.15, 0.50, 1.0] and β = [0.5, 0.4, 0.1].

The reweighting of these two parameter groups follows a visually motivated and task-oriented logic. The initial class-weight setting ***w****_c_* = [0, 0.33, 0.66, 1.0] can be regarded as a simple monotonic linear opacity prior, in which the severity contribution increases from clean to opaque regions. When the vector was adjusted to [0, 0.15, 0.50, 1.0], this adjustment mainly reduces the relative contribution of transparent soiling and enlarges the severity gap between transparent stains and the more occlusive categories. The motivation is that transparent soiling, which is often associated with water-like stains, usually causes weaker visual degradation than dirt- or mud-like occlusions. A similar reweighting logic is applied to the aggregation weights. The initial setting β = [0.6, 0.3, 0.1] gives priority to opacity-aware coverage. And the following adjustment mainly increases the contribution of the spatial term while keeping the local-dominance term unchanged. The reason is that the spatial distribution of soiling is closely related to downstream perception risk, especially for surround-view fisheye images where central and peripheral regions do not contribute equally to perception tasks. Thus, the main static model still remains opacity-dominant but becomes more sensitive to the location-dependent effect of lens soiling. The effectiveness of this reweighting is examined later in Section 5.

Under this formulation, severity becomes a structured representation of global visual degradation that simultaneously encodes category opacity, spatial importance, and local dominance, thereby making the severity representation interpretable and easily generalizable. Eventually, these reweighting attempts should be understood as a task-oriented configuration of the proposed structured severity definition rather than a universal optimum. More importantly, the explicit weight design provides a flexible interface of adapting the severity scale to different downstream perception requirements, camera layouts, and road-use scenarios.

#### 3.2.3. Dual-Head Learning and Consistency Constraint

To make the Structured Severity Score learnable within the network, this paper proposes a dual-head architecture rather than relying on a single image-level regression branch.

Within SDSM, the tile head predicts a tile-level four-class coverage tensor, denoted by $\hat{G}.$ $\hat{G}$ retains local soiling-category and spatial-distribution information and is fed directly into the Severity Aggregator to produce $S_{agg}$. Because $S_{agg}$ is explicitly derived from the predefined score formulation, it represents a global severity estimate with a clear semantic origin rather than an arbitrary learned latent variable.

(13) $$\hat{G}\in R^{8\times 8\times 4}$$

Meanwhile, the global head in SDSM performs global pooling directly on the shared features and uses regression to obtain an image-level severity prediction, denoted as $\hat{S}.$ This branch provides a direct regression path from shared features to global severity, enhancing the learnability and numerical fitting of image-level predictions.

(14) $$\hat{S}=\sigma (w_{g}^{T}v+b_{g})$$

From a modeling perspective, the Tile Head + Severity Aggregator path offers strong local interpretability because it follows an explicit structured severity formulation. Its expressive power, however, is inherently limited by the predefined aggregation rule. The Global Head is therefore intended to capture global feature cues that are difficult to express through tile-level distributions or a fixed score formula alone. It also improves numerical fitting and cross-domain severity ranking under annotation noise.

To prevent the Global Head from drifting away from the tile-level soiling semantics, this study further introduces $L_{\mathrm{cons}}$ to ensure that the global prediction $\hat{S}$ remains consistent with $S_{agg}$. This consistency term anchors the global prediction to the Structured Severity Score space.

(15) $$L_{cons}=\frac{1}{B}\sum_{b=1}^{B}\left|{\hat{S}}_{b}-S_{agg,b}\right|$$

Training under the SDSM framework involves three objectives: learning the tile-level coverage distribution, optimizing the image-level severity regression, and maintaining semantic consistency between the two output paths. The overall objective is therefore written as:

(16) $$L=L_{tile}+\lambda_{glob}L_{glob}+\mu_{cons}L_{cons}$$

where $\lambda_{glob}$ and $\mu_{cons}$ control the relative weights of the global regression term and the dual-head consistency term, respectively.

For the tile branch, we use the mean L1 Loss between the predicted tile-level four-class distribution $\hat{G}$ and the ground-truth tile-level distribution G, thereby learning local soiling coverage and providing reliable structured input to the Severity Aggregator. In the predicted tensor, *B* is the batch size, *C* = 4 denotes the four soiling categories, and *H* = *W* = 8 corresponds to the tile grid resolution. The tile loss is defined as:

(17) $$L_{tile}=\frac{1}{BCHW}{\sum_{b=1}^{B}\sum_{c=0}^{C-1}\sum_{i=1}^{H}\sum_{j=1}^{W}\left||{\hat{G}}_{\left\{b,c,i,j\right\}}-G_{\left\{b,c,i,j\right\}}|\right|}_{1},C=4,H=W=8.$$

For the global branch, we also use an L1 Loss between the image-level prediction $\hat{S}$ and the label S.

(18) $$L_{glob}=\frac{1}{B}\sum_{b=1}^{B}\left|{\hat{S}}_{b}-S_{b}\right|$$

To ensure fair comparison among static variants in later ablation studies, all static models are trained under the same input resolution, backbone, and optimization setting. Unless otherwise stated, the shared settings are 40 training epochs, batch size 32, learning rate $3\times 10^{-4}$, and weight decay $10^{-2}$. Additionally, we use $\mu_{cons}=0.1$ to assess the marginal contribution of the dual-head consistency term.

**Figure 5:**
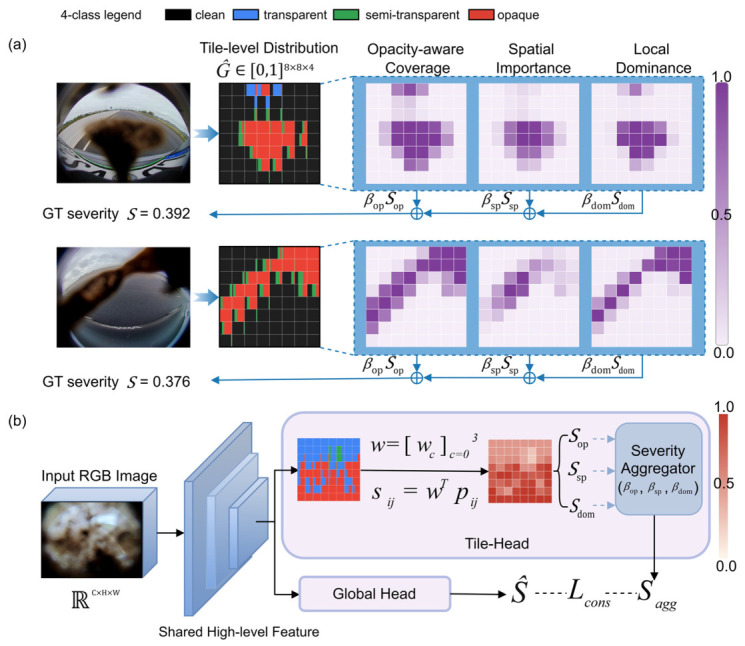
Structured Severity Score and the Structured Dual-Head Static Model. (**a**) Aggregation of tile-level four-class soiling distributions into a global severity score through opacity, spatial, and dominance components. (**b**) SDSM architecture with tile-level distribution prediction, image-level severity regression, and consistency-constrained learning.

Figure 5 illustrates how the Structured Severity Score is constructed and integrated into the SDSM architecture. Top: tile-level four-class soiling distributions are aggregated into a global severity score through the three structured severity factors. Bottom: this formulation is embedded into the SDSM, where a tile head predicts local soiling distributions and a global head regresses image-level severity, with a consistency constraint enforcing semantic alignment between local structure and global severity.

#### 3.2.4. Static Variants Used in the Ablation Study

To isolate the contribution of each structural factor in the Structured Severity Score, this study defines a set of controlled ablation variants for SDSM. These variants are arranged from simplest to most complex, so as to form a progressive evidence chain from area-only reference to the final structured severity formulation.

s, serving as the area-only reference, uses only the overall soiling area ratio without distinguishing among soiling categories.

$S_{\mathrm{op}}$ introduces the class weights $w_{c}$ on top of s so that transparent, semi-transparent and opaque soiling contribute differently to severity. This variant is used to test the effect of category-dependent opacity on severity modeling.

$S_{\mathrm{op}+\mathrm{sp}}$ further adds spatial weighting. A Gaussian spatial map is used so that central image region receives higher importance, allowing us to examine the contribution of spatial importance to severity modeling.

$S_{\mathrm{full}}$ adds the dominance term to $S_{\mathrm{op}+\mathrm{sp}}$, so as to capture the effect of locally concentrated severe soiling on overall perceived degradation. At this point, the complete Structured Severity Score is established.

$S_{\mathrm{full}-\eta 0}$ keeps the overall structure of $S_{\mathrm{full}}$ unchanged but sets the transparent discount coefficient in the dominance term to 0. This variant is used to analyze how transparent soiling contributes to global severity modeling.

$S_{\mathrm{full}-\mathrm{wgap}-\mathrm{alpha}50}$, the main static model used in this paper, rebalances both the class weights and the aggregation weights relative to $S_{\mathrm{full}}$.

$S_{\mathrm{full}-\mathrm{wgap}-\mathrm{alpha}50_{\mathrm{NOCONS}}}$ is derived from the main static model by removing $L_{cons}$. It is used to analyze the impact of consistency constraints on the model’s cross-domain generalization ability.

The main static model established in this section later serves as the frozen static ruler for temporal refinement.

### 3.3. TS-SD for SD-Seq Temporal Supervision

#### 3.3.1. Motivation for TS-SD

Real-world temporal soiling data from in-vehicle cameras has long been scarce, and large-scale collection together with fine-grained manual annotation remains costly. We therefore introduce Stable Diffusion as a controllable source of synthetic supervision. The primary role of TS-SD in this work is to construct temporal supervision for subsequent temporal learning.

#### 3.3.2. Two-Stage Controllable Soiling Synthesis

To obtain controllable soiling layers, the Two-Stage Stable Diffusion (TS-SD) pipeline is adopted. TS-SD decomposes soiling synthesis into two decoupled sub-tasks—Soiling Appearance Modeling and Controlled Composition on Clean Driving Frames—thereby converting an otherwise weakly controllable full-image generation problem into two sequential stages. In the first stage, Stable Diffusion 2.1-base is fine-tuned with LoRA [30] on the strongly annotated WoodScape training set so as to learn the appearance priors of real-world soiling. In the second stage, Stable Diffusion Inpainting is used together with the WoodScape soiling masks as explicit spatial constraints. Under this design, the appearance prior learned in stage 1 is injected into the masked regions, enabling controlled soiling synthesis while keeping the background structure largely unchanged. At inference time, the LoRA weights learned in the first stage—which encode the lens-soiling appearance prior—are loaded into U-Net attention layers of the Inpainting model. To further enhance compositional flexibility, we decouple the clean-frame bank from the mask bank. This allows a single clean background to be paired with multiple soiling layouts, while the same soiling mask can be reused across different road scenes. The resulting design provides reusable synthetic soiling layers for subsequent SD-Seq construction.

#### 3.3.3. Construction of SD-Seq for Temporal Learning

Preliminary inspection indicates that, although TS-SD can generate visually plausible and spatially controllable soiling patterns, the synthesized appearance is not consistently aligned with the class-defined severity semantics. TS-SD is therefore not used as a direct source of single-frame strong supervision. Instead, it is repositioned as a source of temporal mechanism for SD-Seq construction, where the key requirement is cross-frame consistency under the physical prior that the soiling layer remains relatively fixed while the background evolves over time.

**Figure 6:**
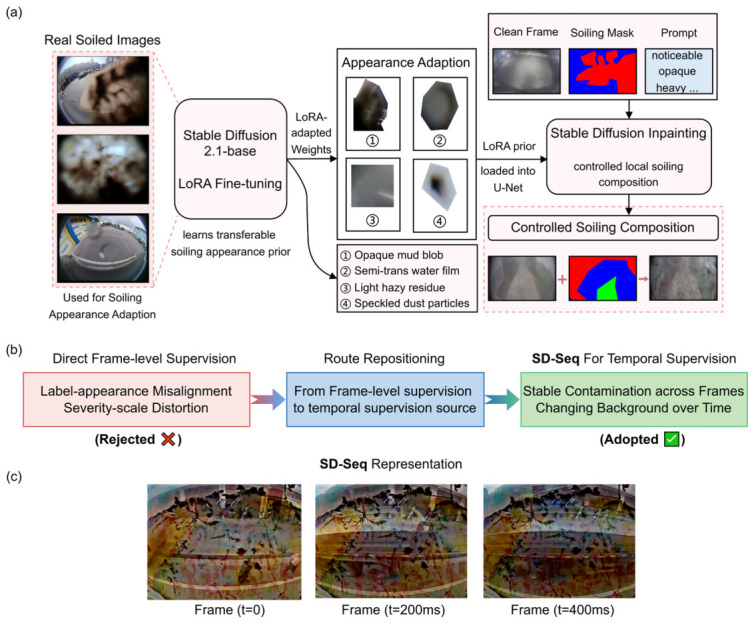
TS-SD pipeline and its role in constructing SD-Seq. (**a**) LoRA-based appearance learning and mask-guided inpainting for controllable synthesis; (**b**) Repositioning TS-SD as the temporal mechanism supervision; (**c**): representative temporal snapshots from an SD-Seq sample, containing scattered opaque mud-like spots and large-area oil-like smear textures.

Following the above role repositioning, we construct SD-Seq for later training. For each clean driving sequence, a single soiling layer generated by TS-SD is assigned and repeatedly overlaid on all consecutive background frames in that sequence. The resulting synthetic sequence preserves temporal variation in the background while maintaining cross-frame spatial consistency and visual stability of the lens-soiling layer. SD-Seq therefore provides controllable temporal mechanism supervision for the subsequent adaptive EMA Module. The final SD-Seq dataset contains 482 synthetic sequences, each fixed at 32 frames, and reuses 173 soiling-layer templates across five surround-view viewpoints, excluding the rear view. Figure 6 illustrates the TS-SD synthesis mechanism, the role repositioning, and the temporal sample format of SD-Seq.

### 3.4. Adaptive EMA Module for Temporal Refinement

Soiling prediction is sensitive to background and illumination changes during vehicle motion, which leads to unstable outputs in continuous video streams. In practical in-vehicle use, such instability affects not only numerical prediction but also alarm logic and downstream assessment of camera availability. The adaptive EMA Module is therefore introduced on top of the established static severity ruler to perform temporal refinement without redefining the severity scale. We use a lightweight, structure-constrained temporal module that adaptively balances smoothing and responsiveness to suppress non-physical jitter.

#### 3.4.1. Formulation of the Adaptive EMA Module

The adaptive EMA Module performs temporal refinement by recursively updating the output around the static anchor using a learned dynamic coefficient $\alpha_{t}$.

(19) $$S_{t}^{temp}=\left(1-\alpha_{t}\right)S_{t-1}^{temp}+\alpha_{t}{\hat{S}}_{t}^{base},\;\;\;\;\alpha_{t}\in [0.05,\;0.40]$$

Here, ${\hat{S}}_{t}^{base}$ denotes the frame-wise prediction of the frozen static ruler at time step *t*, and $S_{t}^{temp}$ denotes the temporally refined output. When the current frame remains consistent with the recent temporal state, $\alpha_{t}$ stays small and the model tends to preserve historical estimates. When a genuine change is indicated, $\alpha_{t}$ becomes larger so that the model can respond more quickly while avoiding excessive lag. Unlike fixed EMA, $\alpha_{t}$ here serves as a dynamic smoothing factor predicted by a mapping network $g_{\theta }$. Its inputs include the visual features of the current frame, the current static-anchor prediction, and the absolute difference between adjacent static-anchor predictions.

(20) $$\alpha_{t}=g_{\theta }(f_{t},\;{\hat{S}}_{t}^{base},\;|{\hat{S}}_{t}^{base}-{\hat{S}}_{t-1}^{base}|)$$

In this formulation, $f_{t}$ provides high-dimensional visual context. ${\hat{S}}_{t}^{base}$ anchors the update to the static severity scale. The difference term $\left|{\hat{S}}_{t}^{base}-{\hat{S}}_{t-1}^{base}\right|$ serves as an explicit change indicator to help distinguish between steady-state and moments of potential change. These three inputs make $g_{\theta }$ behave more like a structurally constrained adaptive filter rather than a replacement for a static severity predictor. The design objective is not to induce large instantaneous fluctuations in $\alpha_{t}$ but to achieve local, low-amplitude yet directionally consistent adaptive adjustment. This behavior is later examined through controlled perturbation experiments.

#### 3.4.2. Frozen-Ruler Training Strategy and Objectives

The adaptive EMA Module is trained on top of the frozen static ruler defined by the main static model. During training, the backbone, tile head, global head, and Severity Aggregator of SDSM are fully frozen, and only the parameters of the temporal module are updated. The frozen static components provide only frame-wise features and static severity predictions. In addition, the backbone remains in evaluation mode to prevent its BatchNorm statistics from being disturbed by the synthetic-sequence distribution.

Training of the temporal module is carried out on short sequences, with outputs computed recursively. This training organization serves two objectives: (1) to preserve the learned static severity ruler, and (2) to introduce temporal stability without contaminating the original static prediction space. With λ = 1.0 and φ = 0.1, the temporal training objective is therefore defined as:

(21) $$L_{temp}=L_{stab}+\lambda L_{keep}+\varphi L_{\alpha }$$

(22) $$L_{stab}=\frac{1}{T-1}\sum_{t=2}^{T}\left|S_{t}^{temp}-S_{t-1}^{temp}\right|$$

(23) $$L_{keep}=\frac{1}{T}\sum_{t=1}^{T}\left|S_{t}^{temp}-{\hat{S}}_{t}^{base}\right|$$

(24) $$L_{\alpha }=\frac{1}{T-1}\sum_{t=2}^{T}\left|\alpha_{t}-\alpha_{t-1}\right|$$

Here, $L_{stab}$ enforces the short-term stability of the temporally refined output and suppresses unnecessary high-frequency fluctuations. $L_{keep}$ keeps the temporally refined prediction close to the frozen static ruler so that the original ranking behavior and numerical severity scale are preserved. $L_{\alpha }$ imposes moderate regularization constraints on the learning behavior of the dynamic smoothing factor, preventing unstable or overly aggressive filtering behavior. This training strategy explicitly separates what the model predicts from how the prediction is temporally stabilized.

#### 3.4.3. Mechanism-Oriented Probing Design for Dynamic $\alpha_{t}$

This section describes the validation process designed to investigate the mechanism of action of $\alpha_{t}$, focusing on both local disturbance response and input dependence.

We first construct a local frame-insertion perturbation test. A stable Level-3 sequence from External Test is used as the base sequence, and a few frames drawn from other Level-3 sequences are inserted at specified positions. The inserted frames are chosen to match camera perspective, road background, and soiling patterns as closely as possible, so that the perturbation remains local and mild rather than causing a global distribution shift. Around the insertion window, this study examines the behavior of the static anchor output, $\alpha_{t}$ and the difference between the adaptive EMA and the fixed EMA. Here, the weighting factor for the fixed EMA is set to the mean of $\alpha_{t}$ learnable by the adaptive EMA Module on the External Test, approximately 0.25.

To analyze input dependence, this paper additionally performs inference-time input ablation by zeroing each of the three inputs to $g_{\theta }$ in turn, without retraining the model. We then compare the perturbation magnitude of $\alpha_{t}$ around the insertion window to assess how each input contributes to the learned dynamic adjustment mechanism.

### 3.5. Evaluation Protocols and Metrics

Because the three datasets differ in supervision format and temporal correlation, this paper adopts dataset-specific evaluation protocols. WoodScape is used for strongly supervised static evaluation, External Test for weakly supervised cluster-level ranking, and OccNuScenes-Dirt for supplementary controlled monotonicity analysis. The temporal refinement effect of the adaptive EMA Module is mainly assessed on External Test.

#### 3.5.1. Strongly Supervised Static Evaluation on WoodScape Test

At the tile level, the model predicts the four-class coverage distribution, which is used to evaluate the regression accuracy of local soiling structures. At the image level, we evaluate both the global-head prediction $\hat{S}$ and the aggregated score $S_{agg}$, which is derived from the tile-level distributions via the Severity Aggregator. Tile-level MAE is used to quantify prediction errors in local coverage regression whereas image-level MAE and RMSE are used to measure global severity fitting error. We further compute $\rho (\hat{S},\;S_{agg})$ and $\rho (\hat{S},\;S_{gt})$, respectively, to evaluate local-to-global within the dual-head architecture and ranking consistency. WoodScape is therefore used to examine whether SDSM can jointly maintain tile-level interpretability, image-level severity fitting, and local-to-global semantic consistency under strong supervision.

#### 3.5.2. Cluster-Level Weakly Supervised Static Evaluation on External Test

Because adjacent frames in External Test sequences often exhibit strong temporal correlations, frame-level evaluation would overestimate the effective sample size and distort statistical conclusions. This study therefore adopts a cluster-level protocol to better reflect model performance under temporally correlated data.

Samples are grouped into clusters according to the video’s temporal structure. The cluster-level prediction is then defined as the median of frame-wise predictions within each cluster. Let $\left\{{\hat{S}}_{i}|i\in C_{k}\right\}$ denote the frame-wise predictions in the k-th cluster. The corresponding cluster-level prediction is:

(25) $${\hat{S}}_{k}^{cluster}=median(\left\{{\hat{S}}_{i}|i\in C_{k}\right\})$$

Cluster-level Spearman correlation is used as the primary metric of cross-domain ranking ability. In addition, this paper reports the standard error (SE) and confidence interval (CI) width to quantify the statistical uncertainty and stability of cluster-level predictions. To estimate confidence intervals of the ranking results, we use bootstrap resampling of clusters. This allows the uncertainty estimates to better reflect the statistical behavior of temporally correlated samples. This protocol ensures that the reported ranking performance is supported not only by point estimates but also by statistically grounded uncertainty quantification under temporally correlated data.

#### 3.5.3. Supplementary Triplet-Monotonicity Evaluation on OccNuScenes-Dirt

OccNuScenes-Dirt provides a supplementary controlled setting for evaluating monotonic severity response. Because it contains multi-level severity triplets that share the same background, it is particularly well suited to assessing whether the model responds monotonically to controlled severity increments. In addition to cluster-level Spearman correlation, we further compute triplet-monotonicity metrics to evaluate the predicted order of severity levels under the same background. Let the model outputs be ${\hat{S}}_{0.1}$, ${\hat{S}}_{0.2}$, and ${\hat{S}}_{0.3}$, respectively. Then, strict triplet accuracy is defined as:

(26) $$Acc_{strict\text{-}triplet}=\frac{1}{N}\sum_{i=1}^{N}I_{strict}^{\left\{i\right\}}$$

(27) $$I=\left\{\begin{array}{c} 1,\;\;\;\;if\;\;{\hat{S}}_{0.1}<{\hat{S}}_{0.2}<{\hat{S}}_{0.3}\\ 0,\;\;\;\;\;\;\;\;\;\;\;otherwise\;\;\;\;\;\;\;\;\;\;\;\;\;\;\;\;\;\; \end{array}\right.$$

Pairwise-triplet accuracy evaluates whether monotonic order holds for the two adjacent severity pairs, 0.1→0.2 and 0.2→0.3.

(28) $$Acc_{pairwise\text{-}triplet}=\frac{1}{2N}\sum_{i=1}^{N}\left(I_{0.1\to 0.2}^{i}+I_{0.2\to 0.3}^{i}\right)$$

(29) $$I_{0.1\to 0.2}=1[{\hat{S}}_{0.1}<{\hat{S}}_{0.2}]$$

(30) $$I_{0.2\to 0.3}=1[{\hat{S}}_{0.2}<{\hat{S}}_{0.3}]$$

These two triplet metrics are primarily used to evaluate the model’s monotonic response capability under degraded conditions within the same scenario, thereby providing an additional test of the robustness of the structured severity representation.

#### 3.5.4. Evaluation Metrics

The evaluation metrics used in this work cover static fitting, ranking consistency, uncertainty quantification, and temporal stability. On WoodScape, Tile MAE measures local coverage regression error, whereas Global MAE and Global RMSE measure image-level severity fitting error. Gap MAE quantifies the discrepancy between the global-head prediction and the aggregated score $S_{agg}$, and Spearman correlation is used to evaluate both local-to-global consistency $\rho (\hat{S},\;S_{agg})$ and prediction-to-label ranking consistency $\rho (\hat{S},\;S_{gt})$. For weakly supervised cluster-level evaluation, bootstrap-based 95% confidence intervals, SE, and CI widths are additionally reported to characterize uncertainty under temporally correlated data.

For temporal refinement, stability is evaluated at the cluster level because the adaptive EMA Module is designed to suppress non-physical short-term fluctuations without redefining the established static severity ruler. MAD and MeanAbsDiff quantify overall and adjacent-frame jitter, respectively, while Variance and Range $\left(p_{95}-p_{5}\right)$ measure the dispersion and effective fluctuation span of predictions within each cluster.

## 4. Datasets Severity Protocol Validity

Before presenting the main experimental results, we briefly validate the functional validity of the two external severity-ranking protocols. Here, we only examine whether these two protocols are functionally consistent with downstream perception degradation.

### 4.1. Validity of the External Test Weak Severity Protocol

The five-level severity scale used in External Test is not an arbitrary weak labeling scheme; it is a visual-degradation-based severity protocol derived from real in-vehicle surround-view images. To test its functional validity, we analyzed more than 70,000 surround-view images containing VRUs and measured downstream pedestrian-detection degradation under different severity levels using an automotive detector. Table 1 shows a clear monotonic trend: higher severity is associated with higher false negative rates and higher blur rates. Levels 4 and 5 are excluded from this specific comparison because their degradation is substantially stronger than that of the first three levels, making the statistics less directly comparable. Overall, the observed downstream deterioration supports the use of External Test as a weak but functionally meaningful severity-ranking protocol.

### 4.2. Validity of the OccNuScenes-Dirt Controlled Severity Protocol

OccNuScenes-Dirt provides a controlled severity ordering through explicit generation parameters. The functional validity of this severity definition is supported by corresponding decline in downstream perception performance. Table 2 summarizes the degradation observed in BEV perception tasks across the three severity levels, as reported by the data team based on the Dirt dataset [31]. The columns corresponding to Dirt-0.1, Dirt-0.2, and Dirt-0.3 report the IoU metrics in the perception tasks. The monotonic decline across these levels supports both the interpretability of the severity ordering and the reproducibility of the protocol definition.

## 5. Experiments and Results

In all tables, the best results are highlighted in bold, and ↑/↓ indicate whether higher or lower values are better. Rows shaded in gray denote the experimental settings with the most prominent overall results across the reported metrics.

### 5.1. Main Static Results of the SDSM

This section presents the static performance of the main model $S_{full\text{-}wgap\text{-}alpha50}$ on the two core benchmarks: WoodScape Test and External Test.

Table 3 shows that the main static model achieves a Global MAE of 0.0217 and a Gap MAE of 0.0145 on WoodScape Test. These results indicate that the global-head prediction remains close both to the ground-truth severity labels and to the aggregated tile-head score. Moreover, the high correlations $\rho (\hat{S},\;S_{agg})$ = 0.9980 and $\rho \left(\hat{S},\;S_{gt}\right)$ = 0.9929 indicate strong local-to-global semantic consistency and reliable global severity ordering.

Table 4 shows that, on External Test, the main static model achieves a cluster-level Spearman correlation of ρ = 0.7876 over 522 clusters, with a 95% confidence interval of [0.7448, 0.8205], SE = 0.0196, and CI width = 0.0757. This result indicates that the main static model maintains strong cross-domain severity-ranking ability under realistic weak supervision, with statistically stable uncertainty estimates under temporally correlated external data.

### 5.2. Ablation on the Structured Severity Score

We next examine whether the transition from area-only statistics to the Structured Severity Score forms a stable evidence chain of improvement. To this end, Table 5 reports a systematic ablation study on External Test.

As shown in Table 5, the area-only reference s reaches only ρ = 0.3174 in cluster-level evaluation, far below all subsequent variants. This indicates that coverage extent alone is insufficient for stable severity ranking across diverse scenarios.

After introducing opacity-aware category semantics, $S_{op}$ raises the correlation sharply to ρ = 0.7020, showing that category-dependent attenuation intensity is a major source of improvement over area-only statistics. Further incorporating spatial and dominance terms yields additional but more moderate gains, with $S_{op+sp}$ and $S_{full}$ reaching ρ = 0.7190 and 0.7236, respectively. These results indicate that spatial importance and locally dominant soiling both contribute to task-oriented severity assessment. At this point, $S_{full}$ constitutes the complete Structured Severity Score.

Setting *η* = 0 in $S_{full\text{-}\eta 0}$ removes the transparent discount from the dominance term and causes ρ to drop sharply to 0.6774, which is even lower than $S_{\mathrm{op}}$ and $S_{\mathrm{op}+\mathrm{sp}}$. This negative result confirms that transparency differences remain essential to a stable severity scale and can offset the benefits of opacity and spatial terms if improperly handled.

The above observations suggest that the contributions of different factors to external generalization are not fully balanced. We therefore rebalance both the class weights and the aggregation weights, leading to the final model $S_{full\text{-}wgap\text{-}alpha50}$. Its correlation on External Test rises to ρ = 0.7876, together with the smallest *SE* and the narrowest CI width, confirming that once the core score structure is established, reasonable weight allocation can further enhance generalization robustness. Furthermore, we remove the consistency term to form $S_{{full\text{-}wgap\text{-}alpha50}_{NOCONS}}$. Compared to the main static model, the decline in performance metrics further demonstrates the necessity of constraining global predictions near the structured severity definition to prevent them from drifting away from tile-based semantics.

In summary, the advantage of the Structured Severity Score does not stem from simply adding individual factors but from organizing opacity, spatial, and dominance into a coherent score structure, together with a balanced interaction between class weights and fusion weights.

### 5.3. Supplementary Controlled-Protocol Validation on OccNuScenes-Dirt

To avoid relying exclusively on External Test, we further evaluate the structured severity representation on OccNuScenes-Dirt under same-background triplets to assess controlled monotonic response. The results are shown in Table 6.

In cluster-level Spearman ranking, s reaches ρ = 0.5234, which is substantially better than its performance on the External Test but still notably lower than all structured variants. This is expected because severity increments in OccNuScenes-Dirt stem from a controlled synthesis protocol, where increasing severity is more directly coupled with increases in coverage and local density. Even in this setting, introducing opacity-aware category semantics raises the correlation to ρ = 0.7564, and adding the spatial term further increases it to 0.7673. The main static model ultimately achieves the best correlation of ρ = 0.7892, indicating that structured scoring remains more reliable than simple area statistics even when area is more directly correlated with severity.

More instructive evidence comes from triplet monotonicity. Unlike cluster-level ranking correlation, which reflects the average ordering performance across clusters, triplet monotonicity directly tests whether the model responds monotonically to increasing severity within the same base scene. For practical lens-soiling monitoring, this is especially important: the system must not only distinguish which condition is more severe but also preserve a consistent directional response as soiling accumulates, thereby ensuring the reliability of alarm threshold triggering, lens usability assessment, and downstream risk evaluation.

From this perspective, the limitation of the area-only severity modeling becomes even clearer in the triplet metrics. For same-background triplets with increasing severity, s reaches only 0.8086 in strict-triplet accuracy and 0.8952 in pairwise-triplet accuracy, both clearly below the structured variants. As opacity-aware semantics, spatial importance, and final weight rebalancing are progressively introduced, monotonic response improves steadily. The main static model reaches 0.9884 in strict-triplet accuracy and 0.9940 in pairwise-triplet accuracy, yielding the best and most stable response under controlled severity increments. These monotonic results show that the model output is more consistent with the task pattern that perceived risk should rise as soiling worsens.

Overall, the results on OccNuScenes-Dirt do not merely replicate the findings on External Test, they provide controlled-protocol validation of the proposed structured severity representation. Even under a degradation protocol whose severity levels are explicitly defined by the synthesis mechanism, the Structured Severity Score and the corresponding main static model remain valid. This further supports the conclusion that the proposed Severity Score possesses cross-domain robustness and is semantically consistent with task-relevant soiling degradation.

### 5.4. Assessment of Single-Frame TS-SD Augmentation

**Figure A1:**
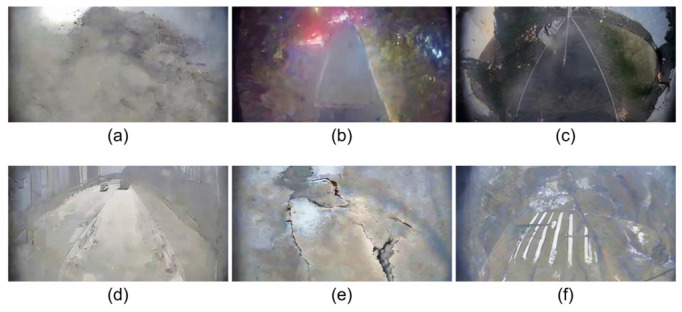
Qualitative examples of single-frame soiling images synthesized by TS-SD. (**a**) Large-area gray dust coverage; (**b**) severe black soiling distributed around the image periphery; (**c**) low-opacity fog-like black soiling; (**d**) low-opacity dust soiling; (**e**) mixed soiling formed by dust and fine black mud streaks; (**f**) flocculent muddy-water soiling.

This section examines whether TS-SD-generated single-frame samples can be directly incorporated into WoodScape training as additional strong supervision for static severity modeling. Representative synthesized samples are provided in the Appendix A (Figure A1).

To test this possibility, we augment the WoodScape training set with TS-SD samples and construct two hybrid models, Real + SD (989) and Real + SD (1260). Here, 989 and 1260 denote the numbers of retained synthetic samples under different filtering strategies, whereas Real-only denotes the main static model trained exclusively on WoodScape. The results in Table 7 show that introducing TS-SD samples does not noticeably worsen numerical regression errors on the WoodScape Test. However, on External Test, the Spearman correlation ρ of both hybrid models is lower than that of Real-only. This indicates that although TS-SD can generate visually plausible soiled images that remain geometrically consistent with real-world scenes, these samples does not directly improve cross-domain severity modeling. Instead, it may interfere with the existing severity scale.

**Figure 7:**
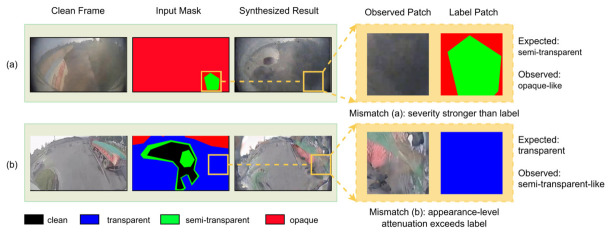
Examples of severity semantic mismatch in TS-SD-generated single-frame samples. (**a**) Opaque soiling effect synthesized from the green-labeled mask region; (**b**) semi-transparent-like soiling effect synthesized from the blue-labeled mask region.

A closer inspection of the generated samples and their corresponding soiling mask labels reveals that the problem does not primarily arise from insufficient texture detail but from a mismatch between the generated appearance categories and the corresponding mask-defined severity labels. Figure 7 provides two representative examples in which regions labeled as transparent in the mask exhibit generated occlusion effects that are visually closer to opaque. This severity semantic mismatch directly distorts the mapping from tile-level coverage to global severity regression. TS-SD is therefore repositioned as a construction route for SD-Seq, where it serves as a source of temporal mechanism supervision for the adaptive EMA Module.

Each case shows the clean frame, the soiling mask, the synthesized result, and enlarged local regions. In some cases, visually stronger attenuation appears in regions labeled as lighter soiling categories.

### 5.5. Main Temporal Stabilization Results on External Test

On External Test, the temporal evaluation of the adaptive EMA Module focuses on two questions: whether it can significantly reduce jitter in continuous predictions, and whether it can preserve the severity ranking established by the main static model while providing such smoothing benefits.

**Figure 8:**
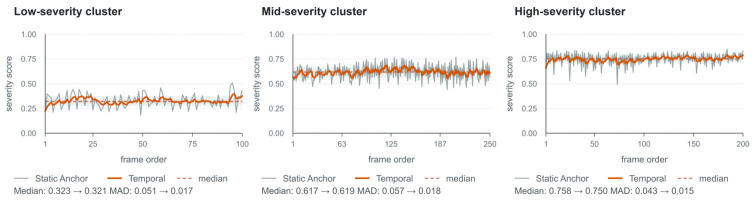
Temporal stabilization results on External Test. Representative frame-wise trajectories for low-, medium-, and high-severity clusters before and after temporal refinement.

Table 8 summarizes the temporal evaluation results of the adaptive EMA Module on External Test. After introducing this module, the Spearman correlation ρ remains relatively stable, while all temporal stability metrics improve substantially. These results show that the adaptive EMA Module can effectively suppress high-frequency temporal fluctuations while largely preserving the static severity anchor. Figure 8 further shows that the adaptive EMA Module consistently smooths short-term fluctuations across different severity levels while preserving the overall severity trend.

The temporal stability gains are not confined to a few extreme sequences but are observed across the vast majority of the 522 independent clusters in External Test. Specifically, 513 of 522 clusters improve in Jitter (MAD), corresponding to a success rate of 98.3%. Moreover, all clusters improve in Jitter (MeanAbsDiff), Variance, and Range $\left(p_{95}-p_{5}\right)$.

To contextualize the learned smoothing behavior of the adaptive EMA Module, we further report fixed-coefficient EMA references using the same static-anchor predictions. These references are not intended to replace the main effectiveness evaluation in Table 8 but to illustrate how different constant coefficients affect the stability–responsiveness trade-off. Since the adaptive EMA Module learns a mean coefficient close to 0.249 on External Test, α=0.25 is used as a matched fixed-EMA reference. Additional fixed values, α=0.20 and α=0.30, are included as nearby alternatives.

The comparison results in Table 9 shows that, under the mostly stable soiling conditions of External Test, the adaptive EMA Module learns a smoothing behavior close to a fixed EMA with α=0.25. This should not be interpreted as a weakness of the adaptive design. Instead, it indicates that the learned coefficient converges to a stable smoothing level when the lens-soiling state remains approximately unchanged. The results with α=0.20 and α=0.30 further illustrate the fixed-EMA trade-off: a smaller coefficient produces stronger smoothing but may cause larger ranking deviation, whereas a larger coefficient better follows the static anchor but suppresses jitter less. The adaptive module learns this trade-off from data rather than requiring manual selection of a global coefficient.

Table 10 further reports the 20 most-improved and 20 least-improved clusters. The comparison shows that the gains of the adaptive EMA Module are concentrated mainly in clusters with high inherent temporal volatility, whereas its effect is naturally limited on clusters that are already relatively stable.

### 5.6. Mechanism Verification of the Learned Dynamic $\alpha_{t}$

The results above show that the adaptive EMA Module significantly improves temporal stability while largely preserving the static ruler. We further examine whether the learned dynamic coefficient can respond to local instantaneous perturbations rather than behaving as a fixed coefficient. To this end, partial-frame insertion probes are constructed around otherwise stable sequences.

Table 11 reports the quantitative results of the local frame-insertion perturbation experiments. The results show a consistent local response of $\alpha_{t}$. In both tested classes, the average $\alpha_{t}$ inside the insertion window is slightly lower than that outside the window. This indicates that, when a short local perturbation appears in an otherwise stable sequence, the adaptive EMA Module moderately reduces the update weight assigned to the current frame and relies slightly more on the historical state. This behavior is consistent with the goal of suppressing transient non-physical disturbances while preserving the stable soiling state. In contrast, fixed EMA applies the same coefficient throughout the sequence and cannot produce such local input-conditioned adjustment. Although the magnitude of the adjustment is small, the response is directionally consistent, and the adaptive output does not fully coincide with the matched fixed-EMA reference within the perturbation window. Their local peak differences reach 0.000300 and 0.000325, respectively, which is inconsistent with a purely fixed-coefficient filtering behavior.

Building on this observation, we further analyze the dependence of the learned $\alpha_{t}$ on different upstream inputs through inference-time input ablation. The results in Table 12 show that removing the visual feature $f_{t}$ produces the largest shift in $\alpha_{t}$, whereas removing the current static-anchor severity summary has a smaller effect, and removing the explicit difference term has the smallest effect. These hierarchical results indicate that the local response of $\alpha_{t}$ depends primarily on high-dimensional visual context rather than being driven directly by low-dimensional numerical signals. At the same time, the static-anchor prediction still provides meaningful task-level semantic information, while the explicit difference term mainly serves as a cue for subtle temporal changes. Taken together, the perturbation and ablation results support the learnability of $\alpha_{t}$ under the proposed structure-constrained formulation.

## 6. Discussion

The goal of this study is not simply to append a temporal module to a static predictor. Rather, it is to establish a feasible static-to-temporal soiling framework that extends single-frame severity assessment towards temporally stable severity assessment under scarce temporal annotation and deployment constraints. The following discussion therefore focuses on three aspects: the practical significance and physical plausibility of temporal stabilization, the methodological value and flexibility of the structured severity representation, and the engineering applicability of the proposed framework in real-world in-vehicle systems.

### 6.1. Trade-Off Analysis

In the lens-soiling assessment, the trade-off between stability and responsiveness is not merely a side effect of filtering but is grounded in the physical prior of the task. This discussion focuses primarily on medium-to-high-opacity soiling patterns that remain relatively stationary with respect to the lens over a short time window, especially those that have a substantial impact on visual perception. By contrast, the road background and illumination conditions continue to change as the vehicle moves. High-frequency fluctuations in single-frame predictions therefore often do not correspond to genuine changes in the soiling state but instead reflect background disturbances, local noise, or short-term observational instability. Under this prior, a moderate sacrifice in instantaneous responsiveness in exchange for smoother and more reliable continuous output is both reasonable and necessary for threshold triggering, status trend monitoring, and maintenance decision support.

However, the purpose of introducing the adaptive EMA Module is not to maximize smoothing unconditionally. Although lens soiling usually remains stable relative to short time windows, sudden adhesion, partial removal, or other short local disturbances may still occur. Therefore, a practical temporal module should suppress frame-to-frame jitter when the soiling state is unchanged, while retaining a bounded response mechanism when local temporal perturbation appears. A fixed EMA can only implement this trade-off through a manually specified global coefficient. In contrast, the proposed adaptive EMA Module learns a dynamic coefficient $\alpha_{t}$ under the frozen-ruler constraint, allowing the update strength to be weakly adjusted according to the current visual context and static-anchor variation.

The experimental results support this design logic from two complementary perspectives. First, on External Test, the adaptive EMA Module significantly reduces instability metrics such as jitter, variance, and range while largely preserving the static severity scale, with the strongest gains concentrated in clusters that originally exhibit the most pronounced fluctuations. This indicates that the module functions as a targeted stability-enhancement mechanism rather than a substitute predictor that achieves superficial smoothness through excessive filtering. In other words, the temporal enhancement proposed here does not attempt to redefine static severity but instead applies structure-constrained corrections to short-term unstable responses in video streams while adhering to the established static severity scale. Second, the partial-frame insertion probes further show that the learned coefficient $\alpha_{t}$ does not behave as a strictly fixed coefficient. Therefore, the advantage of adaptive EMA over fixed EMA should be understood as a learnable and bounded stability-responsiveness mechanism, rather than as a simple attempt to outperform fixed EMA by stronger smoothing.

This conclusion, however, depends on the assumption that soiling remains relatively stable within a short time window. In scenarios involving sudden occlusion, rapid adhesion, or instantaneous removal—where the soiling state changes abruptly—the current adaptive EMA Module may still exhibit response delays. Improving response accuracy under such abrupt events while maintaining an appropriate balance between stability and responsiveness remains an important direction for future research.

### 6.2. Methodological Value and Robustness of the Structured Severity Representation

In SDSM, the Global Head does not operate independently of local structural information; rather, it collaborates with tile-level distributions under the dual-head consistency constraint to model severity. At the same time, soiling severity is decomposed into multiple visual degradation factors and aggregated through category-specific weights and component fusion weights. Consequently, the Structured Severity Score can be tuned to reflect different downstream sensitivities to visual degradation. In this sense, the final static model does not merely output a single-dimensional soiling score; instead, it provides a more structured representation of how different perception tasks emphasize sensitive regions, soiling types, and degradation patterns.

These weight configurations are not arbitrary combinations or heuristic choices; they explicitly encode a structured decomposition of soiling-induced visual degradation. The effects of category-specific transparency, spatial distribution, and local occlusion intensity vary across downstream perception tasks. The weighting system therefore encodes these task-specific differences explicitly into the severity representation, rather than leaving them entirely to the Global Head to fit in latent space.

The significance of this structured representation lies not only in its performance improvement relative to the area-only reference but also in its cross-protocol consistency. External Test primarily validates cross-domain ranking under a real-world weak protocol, whereas OccNuScenes-Dirt tests whether the model responds in a directionally consistent manner under controlled severity increments. Together, these complementary results suggest that, compared with area-only scoring, a structured severity representation integrating transparency differences, spatial distribution, and local dominant effects is more likely to remain stable across heterogeneous external settings.

Beyond the evaluated domains, the structured formulation also provides a practical route for task-oriented adaptation. The current weight configuration can be adjusted according to the dominant soiling characteristics and downstream perception requirements of a target application. For example, scenarios dominated by water-like transparent stains, muddy construction roads, mining roads, or different commercial-vehicle camera layouts may require different emphasis on transparency, opaque occlusion, or task-critical image regions. Under such changes, the same dual-head framework can be retrained with an adjusted Structured Severity Score so that the global prediction remains aligned with the updated severity definition.

At the same time, the proposed score remains anchored to tile-level soiling coverage, which provides a basic and interpretable observation layer. Therefore, even when the detailed weight configuration needs to be adapted to a new domain, the severity representation still retains a coverage-based baseline cue. This coverage anchoring helps reduce the risk that the model relies only on latent image-level shortcuts and makes domain-specific adaptation more transparent than a fully implicit severity regressor.

Although WoodScape provides high-quality strong annotations, its static training scale remains limited relative to typical deep-learning tasks. We therefore further examine the sensitivity of this structured framework to random initialization and data partitioning through multi-seed and k-fold experiments. Under the unified cluster-level evaluation protocol on External Test, the models trained with three random seeds and five k-fold partitions all exhibit a remarkably stable average Spearman correlation ρ. This further indicates that SDSM exhibits good statistical robustness and training reproducibility.

### 6.3. Engineering Value

The scarcity of lens-soiling data limits both the diversity of available temporal supervision and the ability of models to learn reliable priors about short-window soiling evolution under complex conditions. At the same time, limited computing on in-vehicle edge platforms imposes an additional engineering constraint. This is especially true for large commercial trucks, where soiling risk is often higher and the demand for soiling assessment performance is greater, yet deployment resources are typically even more restricted. Under these conditions, approaches based on precise local soiling detection or highly complex temporal modeling are often impractical. A more practical solution should therefore rely on a robust static severity representation and introduce temporal refinement with minimal additional cost.

Following this design logic, ResNet18 is used as a deployment-friendly backbone, and the trained SDSM is frozen before temporal training. The adaptive EMA module then acts only as a stabilizer, refining frame-wise predictions without redefining the established severity scale. TS-SD was used for SD-Seq construction under the physical prior that soiling remains relatively fixed, thereby alleviating the scarcity of temporal training data. Overall, this decoupled design provides a low-cost, compact upgrade path for lightweight soiling models with an established static severity anchor to achieve stable temporal outputs without requiring large-scale collection and ground-truth annotation of real soiling videos.

The semantic mismatch observed in TS-SD during single-frame augmentation may partly stem from the limited dataset size used for LoRA in the first stage. At this stage, Stable Diffusion 2.1-base is adapted using only thousands of images from the WoodScape training set. Although this serves as a reasonable exploratory strategy under constrained conditions, it may not be sufficient for the model to fully learn the severity semantics associated with different soiling categories. If larger-scale and more diverse real-world soiling samples become available in the future, the current semantic mismatch may be alleviated through stronger LoRA adaptation or more extensive fine-tuning of pre-trained diffusion models.

Finally, the static anchor and adaptive EMA Module are functionally decoupled through a frozen design strategy. This design makes upgrade risk more manageable, provides a clearer fallback path, and reduces debugging costs. In addition, monitoring the deviation between static and temporal outputs helps identify abnormal scenarios and potential module failure. The ability to increase responsiveness in highly variable scenarios while strengthening smoothing in stable ones further illustrates a more flexible implementation of the stability–responsiveness trade-off.

### 6.4. Scope of Robustness and Future Work

Although External Test provides a real operational external benchmark, rare or extreme real-world events may still fall outside the current evaluation scope. Examples include rapid adhesion or removal of mud and water, snow or ice soiling, water-flow motion on the lens surface, night glare coupled with lens soiling, cleaning-trigger transitions, and camera-layout changes across vehicle platforms. These cases may introduce different appearance distributions and temporal dynamics from those observed in the current datasets and therefore require dedicated evaluation before deployment in such conditions. This limitation is also consistent with recent robustness studies in autonomous driving, where perception models have been shown to behave differently under natural corruptions, domain shifts, and sensor-related failures than under standard clean benchmarks [32].

As discussed in Section 6.2, the Structured Severity Score formulation provides a practical route for adapting the framework to various new domains by adjusting the severity definition according to target soiling characteristics, camera layouts, and downstream perception requirements. Future work should therefore focus on collecting dedicated abrupt-change sequences, evaluating more diverse open-world soiling patterns, and incorporating uncertainty or time-evolving risk indicators to support cleaning-trigger decisions and reliability-aware camera availability assessment.

## 7. Conclusions

This work goes beyond lens-soiling classification and establishes a static-to-temporal soiling framework that combines interpretability, cross-domain generalization, and temporal stability under scarce real-world data and edge deployment constraints.

At the static level, we propose the Structured Severity Score by explicitly encoding opacity-aware, spatial, and dominance-related factors. On this basis, we establish the Structured Dual-Head Static Model. Results on WoodScape, External Test, and OccNuScenes-Dirt collectively show that the main static model consistently captures soiling-induced visual degradation and exhibits strong cross-domain generalization and ranking capabilities. More importantly, it provides a clearer and more configurable semantic interface for severity modeling across downstream perception tasks.

At the temporal level, TS-SD serves as a source of temporal mechanism supervision. Under the physical prior that the soiling layer remains relatively fixed in camera coordinates while background changes over time, SD-Seq provides a controllable and reusable data foundation for lightweight temporal modules. By freezing the static severity scale, the proposed adaptive EMA Module significantly improves temporal stability in real video streams with minimal parameter overhead, suppressing high-frequency fluctuations while preserving the established severity scale. In addition, the cluster-level evaluation framework used in this work provides a more robust statistical basis for analyzing temporal autocorrelated data.

Overall, the proposed framework provides an interpretable, engineering-friendly, and applicable upgrade path from single-frame soiling models to temporally stable severity monitoring. Future work should focus on improving temporal response under abrupt real-world occlusion events, extending temporal consistency modeling across multiple camera views, and developing supervision mechanisms that more faithfully approximate the physical evolution of soiling.

## Figures and Tables

**Table 1:** Downstream pedestrian-detection degradation across External Test severity levels.

Severity Level	N_Images	Target Detection	Actual Detected	DetectionRate (%)	Fuzzy Detection	FuzzyRate (%)
Level-1	2020	3060	2715	88.7	660	24.3
Level-2	1492	2348	1928	82.1	632	32.8
Level-3	64	204	116	56.9	76	65.5

**Table 2:** Downstream BEV perception degradation across OccNuScenes-Dirt severity levels.

Method	Sensors	Dirt-0.1	Dirt-0.2	Dirt-0.3	Relative Decrease (%)
SimpleBEV	Camera only	38.61	23.42	5.85	84.85%
SimpleBEV	Cam + RADAR	49.84	23.42	5.85	77.91%
SimpleBEV	Cam + LiDAR	49.84	32.86	11.01	71.03%
SimpleBEV	Cam + LiDAR + RADAR	60.43	42.16	16.69	62.57%

**Table 3:** Strongly supervised evaluation on WoodScape Test.

Model	Global MAE ↓	Gap MAE ↓	$\rho (\hat{S},S_{agg})$ ↑	$\rho \left(\hat{S},S_{gt}\right)$ ↑
$S_{full\text{-}wgap\text{-}alpha50}$	0.0217	0.0145	0.9980	0.9929

Note: ↑ and ↓ indicate whether higher or lower values are better.

**Table 4:** Cluster-level weakly supervised evaluation on External Test.

Model	ρ ↑	95% CI	SE ↓	CI Width ↓
$S_{full\text{-}wgap\text{-}alpha50}$	0.7876	[0.7448, 0.8205]	0.0196	0.0757

Note: results are reported under the predefined cluster-level protocol with bootstrap-based uncertainty estimation.

**Table 5:** Ablation of the Structured Severity Score on External Test.

Variant	Opacity	Spatial	Dominance	Transp. Discount	Consistency	Reweighted	ρ ↑	95% CI	SE ↓	CI Width ↓
s							0.3174	[0.2345, 0.3974]	0.0417	0.1629
$S_{op}$	✔				✔		0.7020	[0.6550, 0.7445]	0.0237	0.0895
$S_{op+sp}$	✔	✔			✔		0.7190	[0.6714, 0.7592]	0.0231	0.0879
$S_{full}$	✔	✔	✔	✔	✔		0.7236	[0.6754, 0.7644]	0.0227	0.0890
$S_{full\text{-}\eta 0}$	✔	✔	✔		✔		0.6774	[0.6229, 0.7246]	0.0261	0.1017
$S_{{full\text{-}wgap\text{-}alpha50}_{NOCONS}}$	✔	✔	✔	✔		✔	0.7461	[0.7017, 0.7835]	0.0206	0.0818
$S_{full\text{-}wgap\text{-}alpha50}$	✔	✔	✔	✔	✔	✔	**0.7876**	**[0.7448, 0.8205]**	**0.0196**	**0.0757**

Note: ✔ indicates enabled components; the best result is in bold.

**Table 6:** Supplementary validation on OccNuScenes-Dirt.

Model	ρ ↑	Strict-Triplet Acc($\hat{S}$) ↑	Pairwise-Triplet Acc($\hat{S}$) ↑
s	0.5234	0.8086	0.8952
$S_{op}$	0.7564	0.9427	0.9713
$S_{op+sp}$	0.7673	0.9600	0.9800
$S_{full-wgap-alpha50}$	**0.7892**	**0.9884**	**0.9940**

**Table 7:** Evaluation of mixed training with TS-SD single-frame samples.

Settings	WoodScape MAE ↓	*ρ* ↑	95% CI
Real-only	0.0192	**0.7876**	**[0.7448, 0.8205]**
Real + SD (989)	0.0188	0.7672	[0.7239, 0.8023]
Real + SD (1260)	**0.0187**	0.7106	[0.6590, 0.7525]

**Table 8:** Main temporal stabilization results of the adaptive EMA Module on External Test.

Method	Ranking Preservation	Temporal Stability			
	*ρ* ↑	MAD ↓	MeanAbsDiff ↓	Variance ↓	Range $\left(p_{95}-p_{5}\right)$↓
Static Anchor	**0.7876**	0.0330	0.0870	0.0040	0.1700
+adaptive EMA	0.7829	**0.0160**	**0.0400**	**0.0009**	**0.0770**
△	−0.0047	−0.0170	−0.0470	−0.0031	−0.0930

**Table 9:** Comparison with matched fixed-EMA references.

Method	△*ρ* vs. Static	△MAD	△MeanAbsDiff	△Variance	△Range
Fixed EMA, α=0.20	−0.0051	−0.0185	−0.0496	−0.0029	−0.0930
Fixed EMA, α=0.25	−0.0045	−0.0171	−0.0473	−0.0029	−0.0890
Fixed EMA, α=0.30	−0.0044	−0.0159	−0.0449	−0.0028	−0.0843
Adaptive EMA	−0.0046	−0.0172	−0.0466	−0.0029	−0.0892

**Table 10:** Gain concentration analysis of the adaptive EMA Module on External Test.

Group	Mean Static MAD	Mean Temporal MAD	MeanAbsDiff Reduction ↑	Mean Relative Reduction (%) ↑
Top-20 Clusters	0.0738	0.0276	**0.0462**	**66.4**
Bottom-20 Clusters	0.0254	0.0253	0.00008	1.8

Note: The top-20 and bottom-20 groups are ranked by cluster-wise MAD reduction.

**Table 11:** Results of partial-frame insertion probes.

Setting	Mean $\alpha_{t}$ (Insert)	Mean $\alpha_{t}$ (Non-Insert)	Peak |Adaptive − Fixed|
Single-frame	0.24844	0.24882	0.000300
Two-frame	0.24865	0.24922	0.000325

**Table 12:** Results of approximate inference-time input ablation.

Ablation Mode	Mean Abs $\alpha_{t}$ Shift	Peak Abs $\alpha_{t}$ Shift
No $f_{t}$	0.001094	0.002140
No ${\hat{S}}_{t}^{base}$	0.000225	0.000300
No $\left|{\hat{S}}_{t}^{base}-{\hat{S}}_{t-1}^{base}\right|$	0.000048	0.000143

## Data Availability

Publicly available datasets were analyzed in this study. The WoodScape dataset and the OccNuScenes-Dirt dataset can be obtained from their original public sources. The External Test dataset was prepared by the authors for this study and is not publicly available due to data ownership and project-related restrictions.

## Abbreviations

The following abbreviations are used in this manuscript:

SDSM	Structured Dual-Head Static Model
TS-SD	Two-Stage Stable Diffusion
SD-Seq	Stable Diffusion Sequence
